# Remodelling of the intracardiac ganglia in diabetic Goto-Kakizaki rats: an anatomical study

**DOI:** 10.1186/1475-2840-12-85

**Published:** 2013-06-07

**Authors:** Darius Batulevicius, Thomas Frese, Elmar Peschke, Dainius H Pauza, Vaida Batuleviciene

**Affiliations:** 1 Institute of Anatomy, Faculty of Medicine, Lithuanian University of Health Sciences, Kaunas, Lithuania; 2 Department of Primary Care, Leipzig Medical School, Leipzig, Germany; 3 Institute of Anatomy and Cell Biology, Martin Luther University Halle-Wittenberg, Halle, Germany; 4 Department of Biomedical Diagnostics, Faculty of Medicine, Kauno Kolegija University of Applied Sciences, Kaunas, Lithuania

**Keywords:** Heart, Diabetes, Cardiac ganglia, Nerve, Autonomic nervous system, Neuropathy

## Abstract

**Background:**

Although cardiac autonomic neuropathy is one of major complications of diabetes mellitus (DM), anatomical data on cardiac innervation of diabetic animal models is scant and controversial. We performed this study to check whether long-term diabetic state impacts the anatomy of intracardiac ganglia in Goto-Kakizaki (GK) rats, a genetic model of type 2 DM.

**Methods:**

Twelve GK rats (276 ± 17 days of age; mean ± standard error) and 13 metabolically healthy Wistar rats (262 ± 5 days of age) as controls were used for this study. Blood glucose was determined using test strips, plasma insulin by radioimmunoassay. Intrinsic ganglia and nerves were visualized by acetylcholinesterase histochemistry on whole hearts. Ganglion area was measured, and the neuronal number was assessed according to ganglion area.

**Results:**

The GK rats had significantly elevated blood glucose level compared to controls (11.0 ± 0.6 vs. 5.9 ± 0.1 mmol/l, *p* < 0.001), but concentration of plasma insulin did not differ significantly between the two groups (84.0 ± 9.8 vs. 67.4 ± 10.9 pmol/l, *p* = 0.17). The GK rats contained significantly fewer intracardiac ganglia, decreased total area of intracardiac ganglia (1.4 ± 0.1 vs. 2.2 ± 0.1 mm^2^, *p* < 0.001) and smaller somata of ganglionic neurons. Mean total number of intracardiac neurons in GK rats was 1461 ± 62, while this number in control rats was higher by 39% and reached 2395 ± 110 (*p* < 0.001).

**Conclusions:**

Results of our study demonstrate the decreased number of intracardiac neurons in GK rats compared to metabolically healthy Wistar rats of similar age. It is likely that the observed structural remodelling of intracardiac ganglia in GK rats is caused by a long-term diabetic state.

## Background

Long-term diabetes mellitus (DM) impacts the ultrastructure, cytochemistry and function of neurons and nerves, and leads to a variety of neuropathies [1-4]. Decline in the number of peripheral and central neurons [5-7], decrease of the density of nerve fibres [8], disorganization of the axonal terminals and myelinated nerve fibres [9,10] have been reported in diabetic patients and in animal models of DM. The degenerative effect of DM on neurons and nerves is explained by microvascular insufficiency and biochemical mechanisms [11]. It has been shown that both ultrastructural abnormalities and loss of capillaries result in impaired blood flow of autonomic ganglia and central neurons in DM [12,13]. Additionally, the metabolism of glucose results in the excessive accumulation of free radicals that has a direct neurotoxic effect in DM [14].

One of the major complications of DM is cardiac autonomic neuropathy that damages heart innervation [15]. Clinical studies suggest that cardiac autonomic neuropathy in DM impairs the baroreflex control of heart rate and cardiac tolerance for exercise as well as increases the risk for arrhythmias, silent myocardial ischemia or heart stroke [11]. Although the mechanisms of cardiac autonomic neuropathy in DM are poorly understood, functional studies imply the role of intracardiac ganglia in development of baroreflex circuitry defects and arrhythmias [16,17]. However, anatomical data on the intracardiac ganglia in DM is still scant and controversial. Degenerative ultrastructural changes of the nerve fibres have been demonstrated within atria of diabetic patients [1] and animal models [9]. It was shown that DM significantly decreases the density of cholinergic nerves in the right atrium of rat [17]. On the contrary, other authors found unchanged abundance of cholinergic neurons and even increased density of the cholinergic nerve fibres in the region of sinoatrial node of diabetic mice [18].

Experimental studies have been focused mainly on animal models of type 1 DM [16-18]. One of genetic models of type 2 DM is the Goto-Kakizaki (GK) rat. This model was generated by selective inbreeding of non-diabetic Wistar rats with impaired glucose tolerance over numerous generations [19]. The GK rats develop mild hyperglycemia, glucose intolerance, impaired glucose-induced insulin secretion and other characteristic disorders of type 2 DM early in life [20]. In spite of only mild hyperglycemia in GK rats, both functional and morphological manifestation of diabetic neuropathy have been demonstrated [21]. Goto-Kakizaki rats have been used extensively to study the effects of DM on cardiovascular, renal and endocrine function as well as possible prevention of diabetic neuropathy [20-28]. Up to date there is a lack of anatomical study on the intracardiac ganglia of GK rats, as we did not find any morphological data on the intracardiac ganglia of the type 2 diabetic animal models.

The goal of our study therefore was to check whether the long-term diabetic state impacts the anatomy of intracardiac ganglia in GK rats. We hypothesised that the long-term diabetic state would affect the quantity and size of intracardiac ganglia as well as total number of intracardiac neurons in GK rats.

## Methods

### Animals

All experiments in this study conformed to animal welfare regulations of both German and Lithuanian states and were approved by local committees for animal care and use following the Guide for the Care and Use of Laboratory Animals (NIH Publication No. 85–23, revised 1985). Twelve male diabetic GK rats (inbred, Taconic M&B, Ry, Denmark) were used. Thirteen male Wistar rats (outbred, Schönwalde, Germany) were used as controls. Age of GK rats was 276 ± 17 days (mean ± standard error). Age of control rats was 262 ± 5 days, and it did not differ significantly from GK rats. Rats were fed a standard diet (Altromin 1324, Altromin, Lage, Germany) *ad libitum*. At the moment of sacrifice GK rats weighed 433 ± 5 g, while control rats 512 ± 10 g.

### Blood and tissue sampling

All procedures of blood and tissue sampling were performed on rats that were deeply anesthetised using vaporized isoflurane. Isoflurane (Merck, Darmstadt, Germany) was vaporized at the bottom of a closed glass tank. The rats inhaled the isoflurane and air mixture in the glass tank until being deeply anesthetised. Afterwards, the rats underwent a median laparotomy and thoracotomy. To prevent the formation of blood clots inside the heart, 1000 units of heparin (Liquemin, Roche, Grenzach-Whylen, Germany) were injected into the caudal vein, i.e. equivalent of inferior vena cava in humans. The blood samples were then drawn after puncture of the caudal vein. The caudal vein was cut immediately and the rats were sacrificed by exsanguination still being under deep anesthesia by isoflurane. The hearts with lungs were removed *en block* and rinsed in phosphate buffered saline (PBS) at 4°C. The composition of PBS (pH 7.4) was (in mmol/l): NaCl, 111; Na_2_HPO_4_, 8.1; NaH_2_PO_4_, 1.86. The hearts were then cryo-preserved for 2 hours at 4°C PBS containing 20% sucrose and frozen in the same PBS-sucrose at −20°C. The hearts were stored on dry ice during shipping to Lithuanian University of Health Sciences for further investigation.

### Intact heart preparations

The frozen hearts were thawed by placing them in PBS at 4°C and perfused by injection of PBS. To improve visualization of the neural structures, the atrial walls were distended by injection of warm 20% gelatin solution in PBS. The pericardium, lungs, pulmonary arteries and mediastinal fat were removed from the pressure-inflated hearts. The prepared hearts were pre-fixed for 15 minutes in 4% paraformaldehyde solution in PBS at 4°C (pH 7.4). Following pre-fixing the hearts were rinsed for 30 minutes at 4°C in the PBS containing hyaluronidase (0.5 mg/100 ml, Serva, Heidelberg, Germany) and tetraisopropylphosphoramide (Iso-OMPA, 0.5 mmol/l, Sigma, St. Louis, USA), which inhibits pseudocholinesterase.

**Figure 1:**
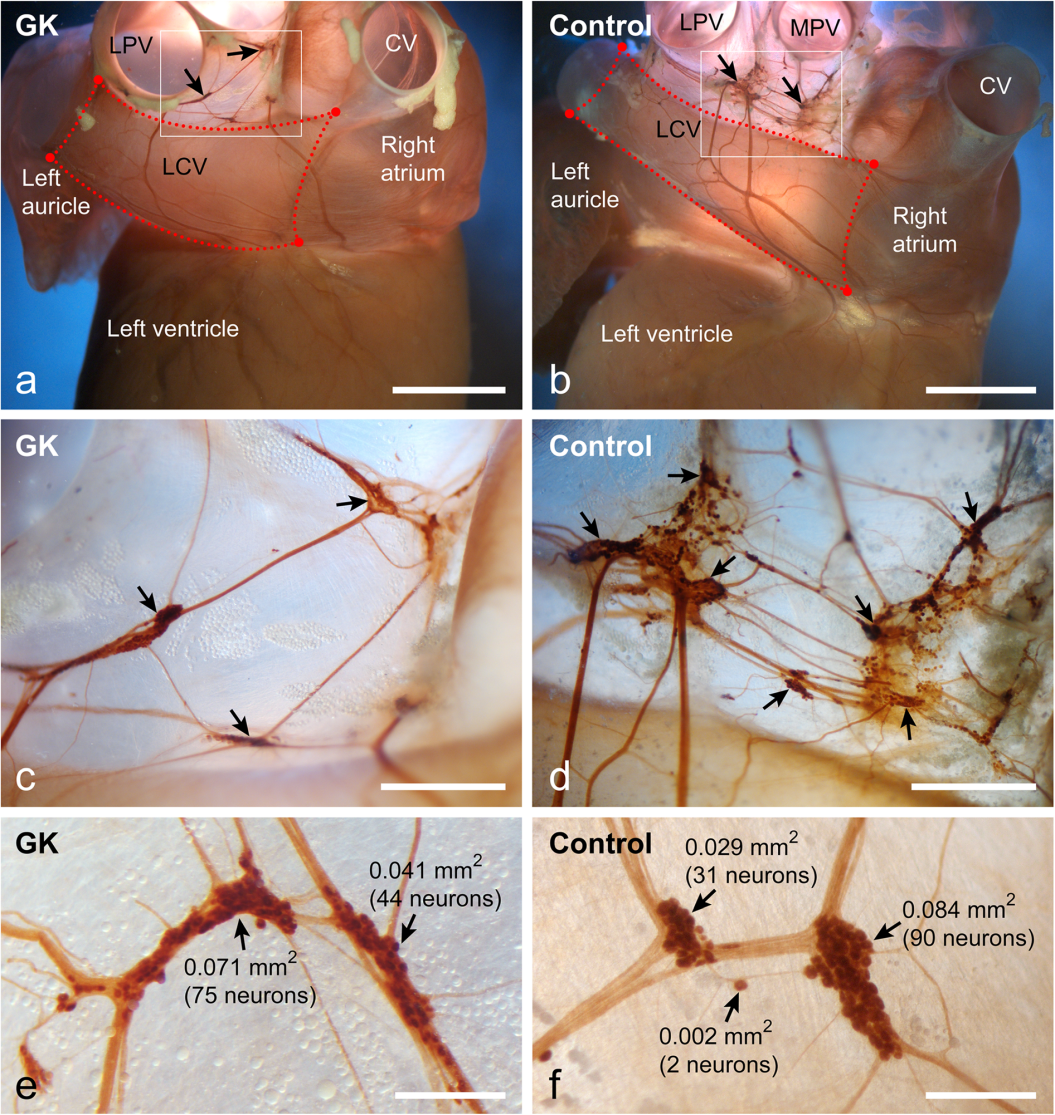
**Intact heart preparations.** Dorsal view of the heart from GK **(a)** and control **(b)** rats demonstrates the intracardiac ganglia (arrows) and intracardiac nerves stained for acetylcholinesterase. Dotted lines demarcate the cardiac region that was used for quantitative analysis of the nerve density. Boxed areas in **a** and **b** are enlarged in panels **c** and **d**, respectively. Note that intracardiac ganglia in GK rat (**c**) are sparse compared to the control rat (**d**). Several intracardiac ganglia (arrows) from GK (**e**) and control (**f**) rats are shown at high magnification with ganglion areas and neuronal numbers indicated nearby ganglia. Neuronal numbers are assessed by linear regression plots as shown in Figure 3a, b. CV, caudal vein; LCV, left cranial vein; LPV, left pulmonary vein; MPV, middle pulmonary vein. *Bars* 4 mm (**a**, **b**), 1 mm (**c**, **d**), 0.4 mm (**e**, **f**).

The nerves and ganglia were stained by the acetylcholinesterase (AChE) histochemistry as described in detail previously [29,30]. The hearts were incubated for 2 hours at 4°C in the AChE staining medium (pH 5.6). The composition of AChE staining medium was (in mmol/l): Na acetate, 60; acetylthiocholine-iodide (Serva, Heidelberg, Germany), 2; Na citrate, 15; CuSO_4_, 3; K_3_Fe(CN)_6_, 0.5. The staining medium was supplemented by Triton-X 100 (Sigma, St. Louis, USA) up to 1% and hyaluronidase (0.5 mg/100 ml, Serva, Heidelberg, Germany). Since the fat on the heart base hindered the staining medium from accessing the neural structures, additional dissection of the fat was performed during staining. Following staining, intact hearts were fixed overnight in 4% paraformaldehyde in PBS (pH 7.4). Heart preparations were examined in distilled water using a stereomicroscope Stemi 2000C (Zeiss, Göttingen, Germany; Figure 1) and preserved in the above para-formaldehyde.

### Analysis of blood glucose and plasma insulin levels

Concentration of blood glucose was analysed with MediSense Precision Xtra glucometer and MediSense Precision Plus strips (both Abbott Diagnostics, Wiesbaden, Germany). Concentration of plasma insulin was analysed by radioimmunoassay using Coat-A-Count kits (DPC, Biermann GmbH, Bad Nauheim, Germany).

### Analysis of total number and area of intracardiac ganglia

The intracardiac ganglia were counted and photographed on AChE-stained intact hearts at 50× magnification of a stereomicroscope Stemi 2000C equipped with an MRC5 camera and Axiovision 4.7.2 software (Zeiss, Jena, Germany). The measurements of ganglion area were done manually by marking the contours of the photographed ganglia with outline spline method of the Axiovision 4.7.2 software. For counting the intracardiac ganglia we subdivided the surface of rat heart into regions of the heart hilum and dorsal left atrial region, as these two areas contain the vast majority of intracardiac ganglia in rat [29].

### Analysis of total number and size of intracardiac neurons

To estimate the neuronal number of intracardiac ganglia and the size of neuronal somata, 50 AChE-stained ganglia from each group of controls and GK rats were excised and covered using water soluble medium Aquatex (Merck, Darmstadt, Germany). The neuronal somata inside these ganglia were counted at 400× magnification using an AxioImager Z1 light microscope (Zeiss, Göttingen, Germany). Based on the ganglion area and the number of ganglionic neurons the linear regression formulas both for controls and GK rats were calculated by SPSS 16.0 software (SPSS Inc., Chicago, USA). The obtained linear regression formulas were then used to assess the neuronal number inside each ganglion (Figure 1e, f). Measurements of the neuronal soma area were performed on 100 neurons selected randomly from each group of controls and GK rats by the same AxioImager Z1 microscope and Axiovision software.

### Analysis of density of epicardial nerves

The stereomicroscopically visible epicardial nerves that supply rat ventricles via the dorsal wall of the left atrium and sinus of the left cranial vein were photographed using a stereomicroscope Stemi 2000C, Axiocam MRC5 camera and Axiovision 4.7.2 software (Zeiss, Jena, Göttingen, Germany). The AChE-stained nerves were outlined on the computer screen, and the percentage of outlined pixels in the digital image was estimated by the histogram method of Photoshop 7.0 software (Adobe Systems, USA).

### Statistical analysis

Results are presented as mean ± standard error. Statistical comparisons between GK and control groups were performed using Mann–Whitney U two-tailed test (SPSS 16.0, SPSS Inc., Chicago, USA). Significance was accepted at *p* < 0.05.

## Results

### Blood glucose and plasma insulin levels

**Figure 2:**
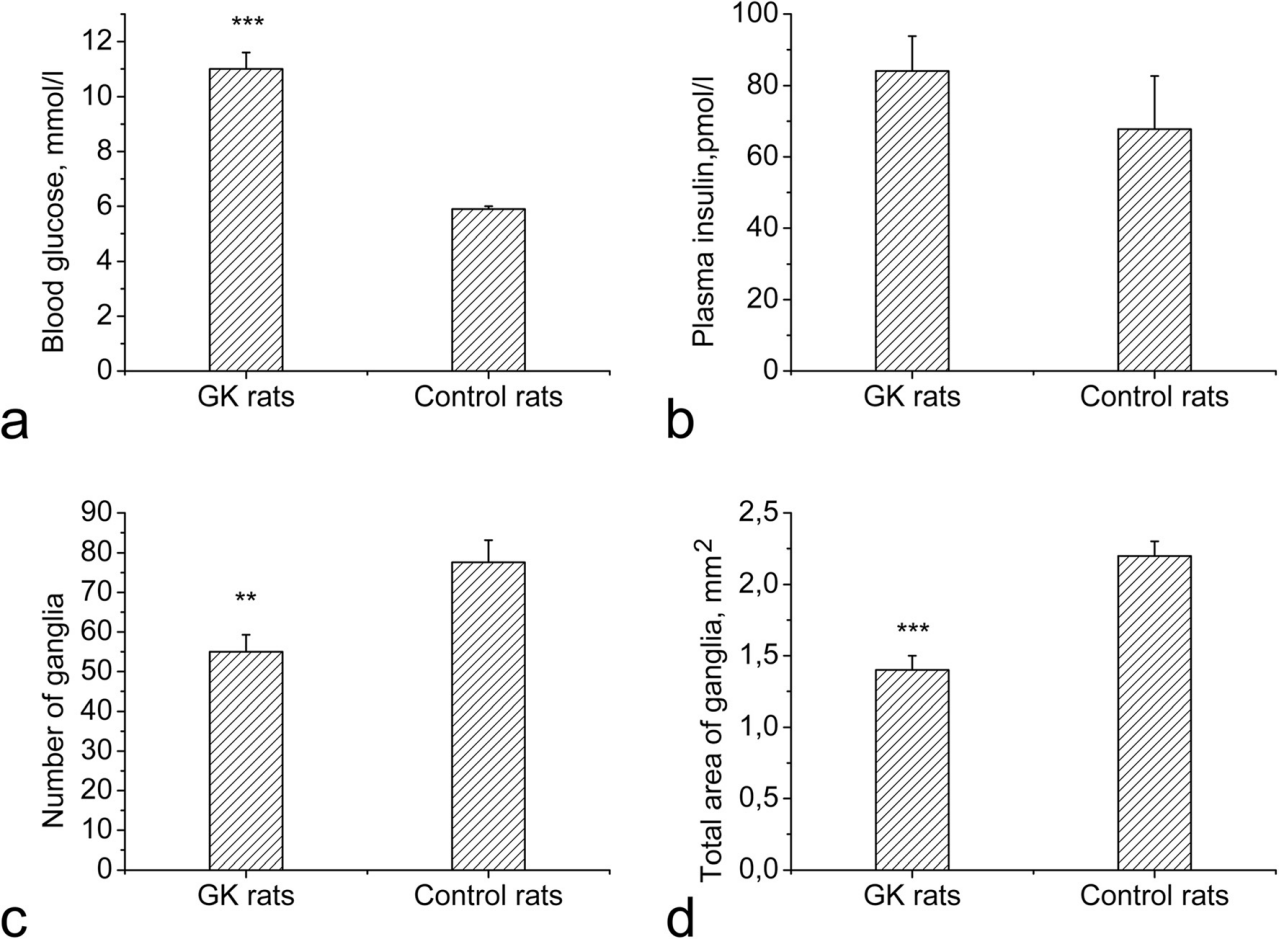
**Blood glucose and plasma insulin levels as well as number and total area of intracardiac ganglia.** Goto-Kakizaki rats exhibit significantly elevated blood glucose levels compared to controls (**a**), while the plasma insulin level is not significantly different between two groups (**b**). Both the number of intracardiac ganglia (**c**) and the total area of ganglia (**d**) are significantly decreased in GK rats compared to controls.**p < 0.01, ***p < 0.001.

Blood glucose level in control rats was 5.9 ± 0.1 mmol/l. It was significantly increased to 11.0 ± 0.6 mmol/l in GK rats (*p* < 0.001; Figure 2a). Plasma insulin level in control rats was 67.4 ± 10.9 pmol/l, but it did not differ significantly from plasma insulin level 84.0 ± 9.8 pmol/l of GK rats (*p* = 0.17; Figure 2b).

### Total number and area of intracardiac ganglia

Control rats contained 78 ± 6 intracardiac ganglia while this number was reduced to 55 ± 4 in GK rats (*p* < 0.01; Figure 2c). Significant loss of intracardiac ganglia and considerably reduced total area of intracardiac ganglia were found in the heart hilum (Table 1). Loss of intracardiac ganglia in the dorsal atrial region was not significant (*p* = 0.06), but total area of intracardiac ganglia in this region was significantly reduced as well (Table 1). Total areas of intracardiac ganglia in GK rats and controls were 1.38 ± 0.06 mm^2^ and 2.22 ± 0.10 mm^2^, respectively (*p* < 0.001; Figure 2d).

**Table 1:** **Number and total area** (**in mm**^**2**^) **of intracardiac ganglia stained for acetylcholinesterase** (**mean** ± **standard error**) **in GK** (**n** = **12**) **and control** (**n** = **13**) **rats**

**Cardiac region**	**GK rats**	**Controls**
*Number of ganglia*		
Heart hilum	39 ± 4	56 ± 5*
Dorsal atrial region	17 ± 1	21 ± 2
Overall	55 ± 4	78 ± 6**
*Total area of ganglia*		
Heart hilum	0.79 ± 0.05	1.45 ± 0.10***
Dorsal atrial region	0.59 ± 0.04	0.79 ± 0.05**
Overall	1.38 ± 0.06	2.22 ± 0.10***

*p < 0.05, **p < 0.01, ***p < 0.001.

### Total number and size of intracardiac neurons

**Figure 3:**
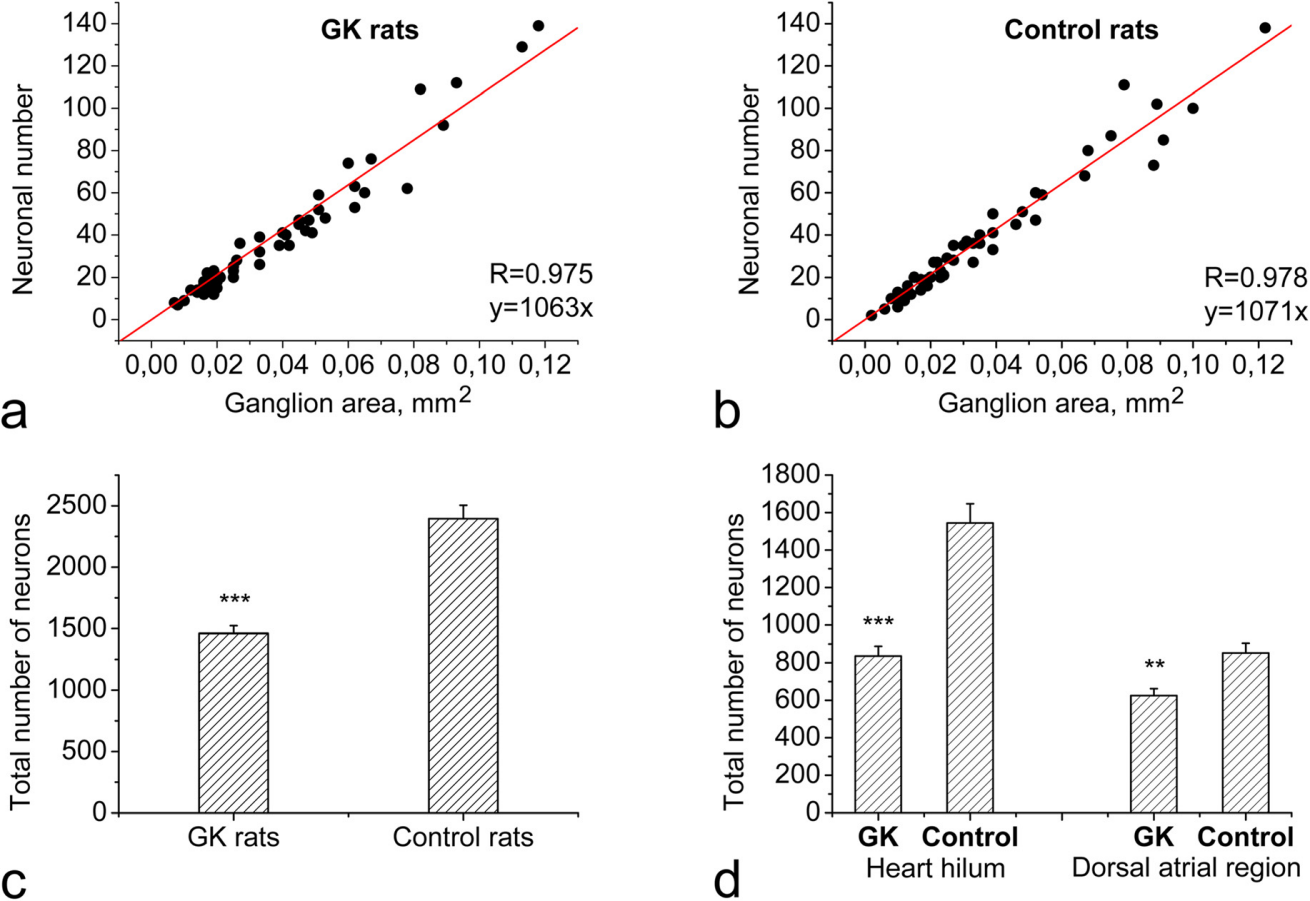
**Assessment of the total number of intracardiac neurons.** Correlation between the ganglion area and neuronal number is shown for the heart of GK (**a**) and control (**b**) rats. Note the strong correlation between the ganglion area and the neuronal number in both groups. Total neuronal number per heart (**c**) and neuronal numbers in both the heart hilum and the dorsal atrial region (**d**) are significantly decreased in GK rats compared to controls. **p < 0.01, ***p < 0.001; R, correlation coefficient. x, ganglion area in mm^2^; y, number of ganglionic neurons inside ganglion.

Strong correlation between the ganglion area and neuronal number was found both for GK rats and controls (Figure 3a, b). Linear regression revealed that the number of neurons inside intracardiac ganglion of GK rats and controls may be correspondingly approximated by linear equation formulas N = 1063 × S and N = 1071 × S, where N is neuronal number and S is ganglion area in square millimetres (Figure 3a, b).

**Figure 4:**
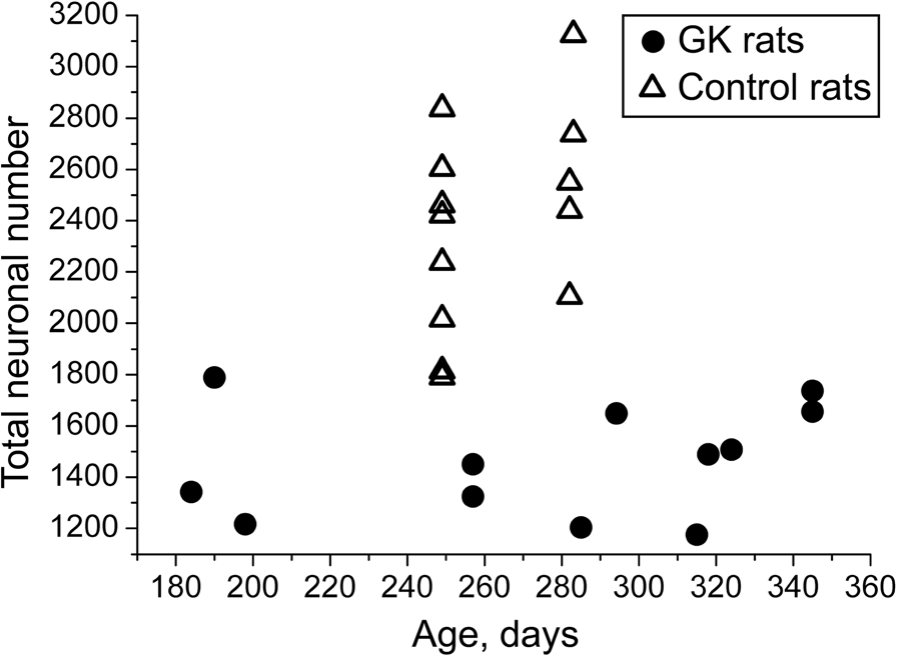
**Total number of intracardiac neurons plotted against age** (**in days**) **of GK** (**n** = **12**) **and control** (**n** = **13**) **rats**. Note that the decrease in total neuronal number in GK rats is not gradual in the investigated age range.

Total number of intracardiac neurons in GK rats was significantly decreased in respect to control animals (1461 ± 62 vs. 2395 ± 110, *p* < 0.001; Figure 3c). Heart hilum of GK rats and controls accumulated 836 ± 52 and 1544 ± 103 neurons, respectively (*p* < 0.001; Figure 3d). The dorsal atrial region of GK rats and controls accumulated 625 ± 37 and 851 ± 52 neurons, respectively (*p* < 0.01; Figure 3d). The decrease of the total number of intracardiac neurons was not gradual in the investigated age range of GK rats (Figure 4). In addition, the area of neuronal soma in GK rats was significantly decreased compared with control rats (810 ± 19 μm^2^ vs. 934 ± 23 μm^2^, *p* < 0.001).

### Density of epicardial nerves

**Figure 5:**
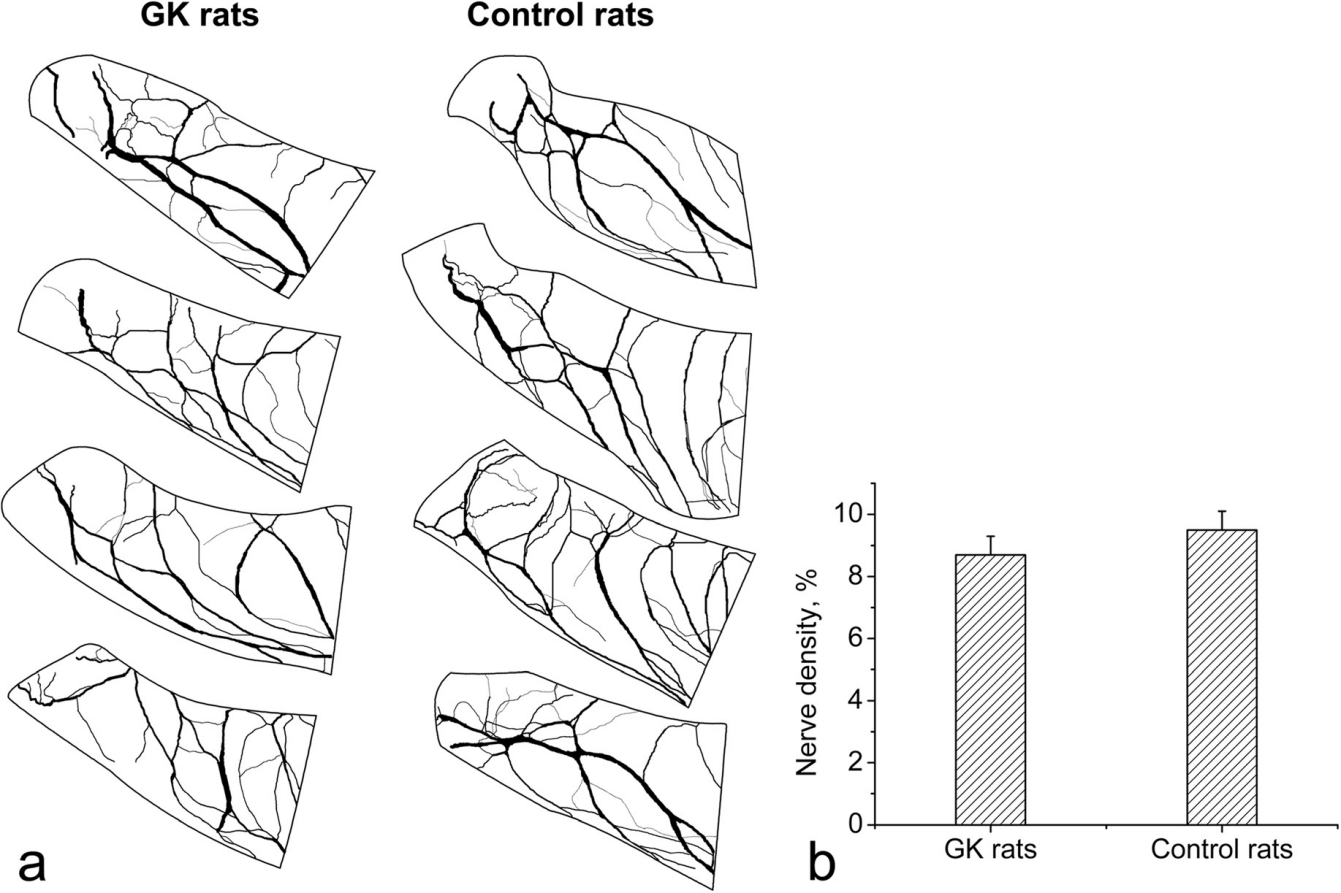
**Density of epicardial nerves.** Drawings of epicardial nerves in the dorsal left atrial region stained for acetylcholinesterase from four GK and four control rats (**a**) demonstrate the similar density of nerves between GK rats and controls. Density of epicardial nerves (expressed as percentage of area covered by the nerves) in the dorsal left atrial region (**b**) does not differ significantly between GK rats and controls.

Density of epicardial nerves in GK rats was 8.7 ± 0.6% while in controls it was 9.5 ± 0.6% (Figure 5). The density did not differ significantly between the two groups (*p* = 0.23; Figure 5). Although quantitative analysis of density of nerves was limited to the epicardium of the dorsal wall of the left atrium, density of nerves in other epicardial regions was evidently similar between GK rats and controls.

## Discussion

The results of our study demonstrate that adult GK rats aged approximately nine months exhibit marked hyperglycemia, but no significant hyperinsulinemia. Hearts of GK rats contain fewer ganglia than control Wistar rats of similar age, and the area of intracardiac ganglia is significantly decreased in GK rats. Total number of intracardiac neurons in GK rats is about 39% less than in controls. Moreover, the size of soma of intracardiac neurons is decreased by 13% in GK rats. Hence, we consider that significant structural remodelling of the intracardiac ganglia is characteristic for adult GK rats. This remodelling of the intracardiac ganglia in GK rats is likely to be due to metabolic disorders associated with the long-term type 2 DM. Our study is a first demonstration of the structure of intracardiac ganglia in type 2 diabetic rats.

Although the functional impairment of cardiac autonomic innervation in DM has been extensively studied [31-37], there is still very little data on anatomy of intracardiac ganglia of diabetic animal models. Reduction of size of both intracardiac ganglia and intracardiac neurons has been reported in type 1 diabetic mice [16,18]. Our study demonstrates that decrease of the total number of intracardiac neurons as well as reduction of size of both intracardiac ganglia and intracardiac neurons is characteristic for adult type 2 diabetic rats. Finding of the decreased number of intracardiac neurons in GK rats is new, because no other studies have attempted to estimate the total number of intracardiac neurons in this animal model of DM. Previous study by Mabe and Hoover [18] on type 1 diabetic mice reported no difference in abundance of intracardiac neurons after 4 months of DM [18]. Although the exact cause of different findings between our study and the report by Mabe and Hoover [18] is unknown, this disparity might be explained by 1) different kind of DM and longer exposure of animals to DM in our study, 2) different animal model used, and 3) different methodological approach to quantify the intracardiac nerve cells between the studies. We attempted to quantify the vast majority of rat intracardiac ganglia, because they were readily visible on the surface of intact heart preparations. In contrast, Mabe and Hoover [18] counted neurons in serial cryostat sections of mouse atria. It seems that cryostat sectioning of very thin atria of mouse may provide significant underestimation of the total number of intracardiac neurons. Indeed, the estimate of total neuronal number of intracardiac neurons in control mice reported by Mabe and Hoover [18] is more than five times less compared to that reported by other authors who counted intracardiac neurons in whole-mount preparations of mouse atria [38].

Loss of neurons has been previously reported in retina [7], intestines [39-41] and stomach [42] of type 1 diabetic rats as well as in retina [43,44] and hippocampus [6] of type 1 diabetic mice. The extent of neuronal loss in GK rats identified in our study is comparable to that reported in intestines and stomach of type 1 diabetic rats [39-42], and even higher compared to retina of diabetic mice [43,44]. However, the molecular mechanism by which the nerve cells develop structural abnormalities and neuronal number decreases is not yet clear. Generally, it is assumed that oxidative stress, exposure to advanced glycation end products, depletion of neurotrophic factors or activation of polyol pathway might contribute to the neuronal death [43,44]. Activation of the polyol pathway has been shown to account for the induction of early neuronal apoptosis of retinal ganglion cells [12]. Thus, it would be tempting to look for apoptosis of intracardiac neurons of GK rats in future.

We found similar density of major nerves in the left atrial epicardium between GK rats and controls. Previous studies have found reduced [45-47], unchanged [33] or increased [18] density of intracardiac nerves in diabetic animal models. This diversity of findings regarding density of intracardiac nerves may be attributed to the differences in the functional kind of nerves and cardiac region analysed by previous studies. It is generally accepted that long-term DM results in loss of peptidergic sensory nerve fibres [45,46]. However, it is also supposed that DM does not affect the density of adrenergic intracardiac nerves significantly [17,18,33]. Decrease of ventricular but not atrial innervation density by neuropeptide Y-positive nerves was observed in rats after long-term DM [47]. Density of cholinergic nerves has been found as significantly decreased [17] or increased [18] in right atrium of diabetic animal models. Taken together with the above findings by other authors, our study demonstrates that the major nerves in the left atrial epicardium of adult rat remain intact after long-term type 2 DM. These major nerves in the left atrial epicardium presumably include both adrenergic and cholinergic nerve fibres with the high proportion of the fibres of adrenergic kind [38]. Since AChE histochemistry stains both cholinergic and adrenergic nerves [48], our technique did not allow us to discriminate between those two kinds of fibres. Therefore, staining of both cholinergic and adrenergic nerves in different cardiac regions analysed could explain the differences of findings between our study and other authors [17,18]. Future immunohistochemical studies for cholinergic, adrenergic and peptidergic markers should ascertain the effect of type 2 DM on regional distribution of intracardiac nerve fibres in GK rats.

With respect to significance of the decreased number of intracardiac neurons in DM, our findings support the idea that intracardiac ganglia might be involved in parasympathetic dysfunction observed in diabetic patients [11] and animal models [16,49]. Since the intracardiac ganglia represent an efferent limb of baroreflex circuitry, the decreased number of intracardiac neurons may impair the parasympathetic efferent control of cardiac tissues and could result in the diabetes-induced baroreflex deficit [16]. Additionally, the decreased number of intracardiac neurons may result in the defects of balance between parasympathetic and sympathetic tone on the heart. Autonomic imbalance characterized by the impairment of parasympathetic function and a relative increase of sympathetic function is one of serious complications that appear early in DM [35,50]. Thus, further studies are essential to find measures preventing negative effect of DM on intracardiac ganglia.

The following limitations of our study should be considered: 1) We did not perform functional studies to test if the parasympathetic function of intracardiac ganglia and intracardiac nerves is preserved in GK rats; 2) We did not perform electron microscopic analysis of intracardiac ganglia and immunohistochemical characterization of adrenergic, cholinergic and peptidergic nerve fibres; 3) We did not estimate the total number of intracardiac neurons in newborn and juvenile GK rats to determine the timing of onset of the neuronal loss.

## Conclusions

We conclude that GK rats with the age of about nine months exhibit shrinkage of intracardiac ganglia and a decrease in the total number of intracardiac neurons. Whether and how this structural remodelling of intracardiac ganglia results in functional deficits of cardiac innervation in GK rats remains to be elucidated.

## Abbreviations

AChE: Acetylcholinesterase; DM: Diabetes mellitus; GK: Goto-Kakizaki; PBS: Phosphate buffered saline.

## Competing interests

The authors declare that they have no competing interests.

## Authors’ contributions

DB designed the study, performed the anatomical research, interpreted the data and wrote the manuscript. TF provided samples for the study, performed the biochemical research and initiated the study. EP contributed to the study concept, provided the animals and materials for tissue sampling and biochemical research. DHP revised critically the manuscript and provided materials for anatomical research. VB analysed data, performed quantitative analysis and edited the manuscript. All authors reviewed and approved the final version of manuscript.
